# Attitudes and Perceptions of Neuroanatomy Lecturers towards the Relevance of Neuroanatomy in the Medical and Physiotherapy Curriculum at Northeast Brazilian Health Profession Schools

**DOI:** 10.1007/s40670-025-02631-1

**Published:** 2026-01-21

**Authors:** João Henrique Ferreira França, Maria Clara Santana Lira, João Paulo De Oliveira Jeronimo Rodrigues, Reynaldo de França Souza, Reynaldo de França Souza, Tainá Ottoni Borges Igreja Ramos Brandão, André de Sá Braga Oliveira

**Affiliations:** Department of Morphology, Federal University of Paraiba, João Pessoa, Paraíba, Brazil

**Keywords:** Neuroanatomy education. neurophobia. medical education. physiotherapy education. teaching strategies

## Abstract

Neuroanatomy is widely recognized as a challenging discipline, often leading to neurophobia among students and disinterest in neurology among healthcare professionals. The current study aimed to evaluate the perceptions and teaching approaches of neuroanatomy professors in Medicine and Physiotherapy programs at universities of Paraíba, Brazil, in order to determine whether the lecturers’ strategies could be a contributing factor to the low engagement in neurosciences. A cross-sectional study that utilizes a quantitative method of data collection with qualitative enhancement was performed with neuroanatomy lecturers from universities of Paraiba, Brazil. They were invited to complete an online questionnaire. A total of 16 professors (8 from Medicine course and 8 from Physiotherapy course) from 11 institutions were interviewed. In our sample, lecturers teaching in Medicine tended to hold higher formal qualifications compared to those teaching in Physiotherapy. Despite the difference in formal qualifications, professors of both courses identify themselves as proficient in teaching neuroanatomy. Concerning to practical lessons, a low use of cadaveric specimens, anatomical models and dissection was observed in professors of both courses.The most frequently cited literature was *Functional Neuroanatomy* by Angelo Machado. Additionally, 87.5% of the professors reported using supplementary materials, such as YouTube videos, to enhance learning. Although there is a growing interest in digital tools, resistance to curricular modernization remains a challenge, especially among professors of Medicine. Moreover, low use and availability of cadaveric specimens in practical training, limited instructional time and limited access to modern technologies can compromise the quality of neuroanatomy learning, leading to neurophobia and low engagement in the neurosciences field for future health professionals. Investments in body donation programs, pedagogical innovation, interdisciplinary integration and infrastructure could optimize teaching and learning of neuroanatomy in the population studied.

## Introduction

The clinical practice of healthcare professionals is based on theoretical and practical knowledge acquired during academic training, providing the foundation to promote patient health. Therefore, there is a need to integrate the teaching of basic sciences, such as anatomy, with clinical practice, allowing healthcare professionals to make coherent decisions, more precise diagnoses, and safe surgical interventions [1]. For the area of neurology and/or neurosurgery, the literature demonstrates that mastery of neuroanatomy is essential for the success of neurosurgical procedures and for advances in surgical techniques and diagnostic accuracy. It is also fundamental in outpatient neurology, where correlating anatomical structures with motor, sensory, and cognitive signs supports precise clinical evaluation [2].

In anatomy teaching, students often perceive the nervous system as complex and difficult, requiring more study and greater ability to integrate abstract knowledge, making the learning process more difficult and time-consuming [3]. Furthermore, the complexity of nervous system structures and the difficulty in visualizing abstract connections, such as neural pathways, make learning challenging. In addition, the scarcity of anatomical specimens and efficient teaching strategies can contribute to students’ lower affinity for the content [4]. International research has shown that students often struggle to integrate neuroanatomy with neurophysiology, neuroimaging, and clinical reasoning, resulting in fragmented learning across different educational systems. These findings suggest that difficulties in connecting structural knowledge to functional and clinical applications are part of a broader global pattern, not limited to local educational contexts [5].

The perceived complexity of neuroscience can lead to neurophobia, characterized by disinterest in neurology, reduced confidence in neurological concepts, and decreased engagement among students and healthcare professionals [6]. This issue is reinforced by a predominantly theoretical approach that limits opportunities to apply neuroanatomy in clinical contexts and makes concepts less tangible [7]. In response, professors, who often perceive the content as inherently complex, must convey abstract concepts and are encouraged to adopt strategies that facilitate learning, guiding methodological choices toward approaches that make neuroanatomy more accessible and meaningful to students. Such strategies may also help reduce students’ aversion to the subject and mitigate neurophobia [4].

Another challenge faced by professors is the scarcity of adequate resources, such as cadaveric specimens and the absence of neuroimaging equipment, which enable proper neurosciences teaching. For this reason, classes are limited to theoretical and often unilluminating explanations that result in a decrease in student interest, as well as an overload on faculty who need to stay updated with new studies, diagnoses, and therapeutic approaches [8]. These challenges underscore the need to anchor neuroanatomy instruction in established learning theories. In this regard, Cognitive Load Theory offers an appropriate framework, as it addresses how complex information can overwhelm learners and emphasizes strategies that optimize cognitive processing. Incorporating this perspective supports the development of approaches that make learning more efficient and meaningful [9].

Thus, the present study proposes to evaluate the attitudes and perceptions of professors regarding the teaching and learning of neuroanatomy among medical and physiotherapy students in the Brazilian Northeast. These courses were selected because, according to their pedagogical curricula, they allocate the greatest workload to the study of the nervous system, and neuroanatomy constitutes a core subject within the basic cycle of health sciences programs, typically offered within a single semester. This evaluation seeks to identify the difficulties reported by faculty and students in order to support the development of strategies that enhance the teaching of this content.

## Methods

**Fig. 1:**
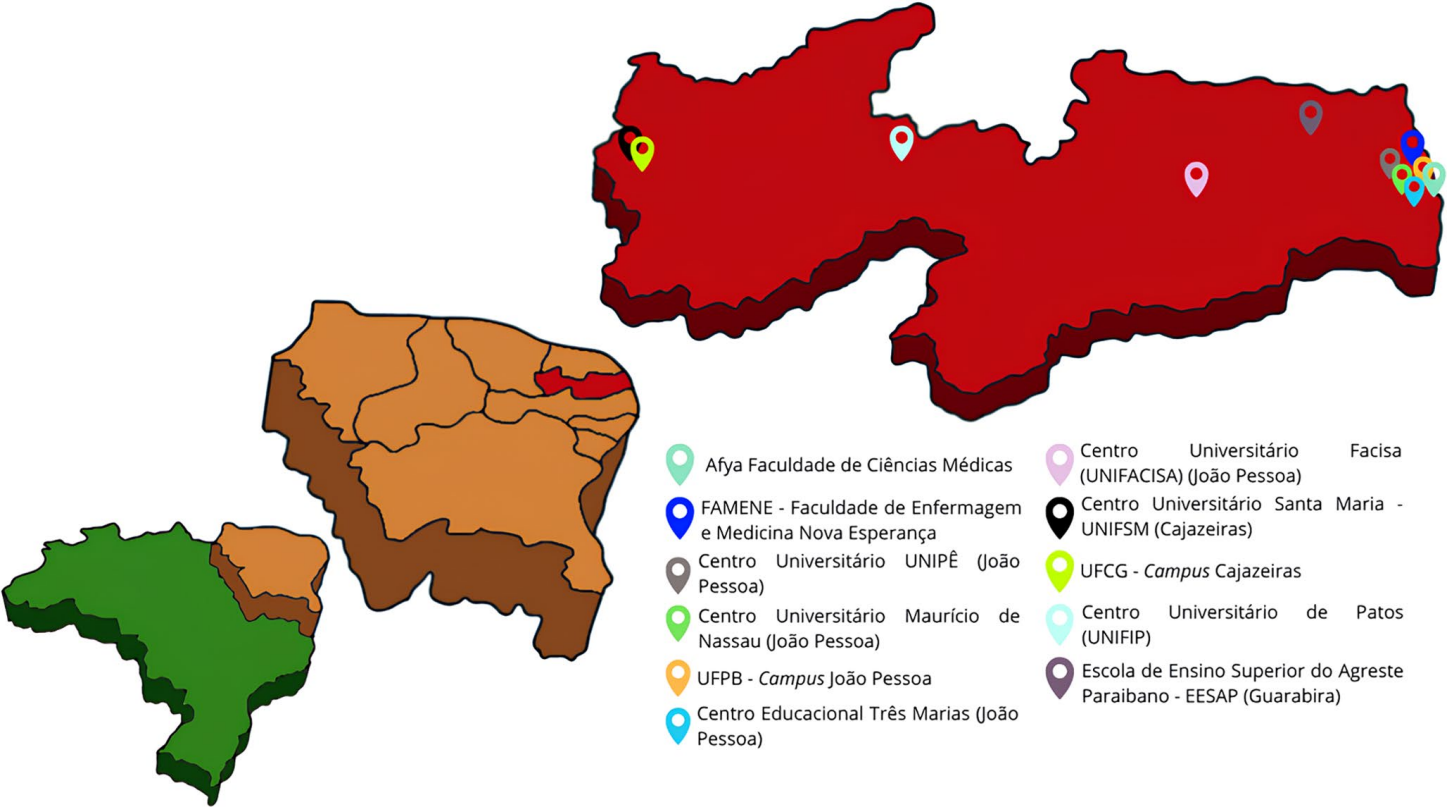
Map of Paraíba (in red), a state from Northeast (in beige) Brazil (in green), indicating the location of the universities included in this study

This cross-sectional study employed a quantitative data-collection approach with a descriptive component and was designed as an exploratory pilot investigation. It was conducted across 11 universities in the state of Paraíba, located in northeastern Brazil, which has an estimated population of approximately 4 million inhabitants (Fig. 1). Faculty members from Medicine and Physiotherapy courses who taught neuroanatomy for more than 20% of the workload of the respective curricular component were included.

The professors responded to a quantitative questionnaire that included agreement-based questions (agree/disagree), matrix-scale items, four-level categorical questions, and open-ended responses. The qualitative component consisted of open questions in which professors described, for example, the bibliographic references they used. These narrative answers were later compiled and summarized descriptively to complement the quantitative data. The questionnaires were adapted and validated by independent academic and statistical consultants from previous studies [10–14]. Biographical data, academic background, teaching experience, and perceptions of neuroanatomy’s relevance were collected via questionnaires, administered online (Google Forms^®^). Participants received the Free and Informed Consent Form (FICF) with study details and criteria, and online forms were distributed via email or WhatsApp. The study was approved by the Research Ethics Committee of the Health Sciences Center of the Federal University of Paraíba (CCS-UFPB), CAAE 79608824.8.0000.5188, with consent and approval from all participating institutions, following the ethical principles of Resolution 466/12 of the National Health Council for research involving human beings (Brasil, 2012). Data were recorded using Word and Excel for analysis, and statistical analysis was performed with Jamovi 2.6 [15]. The χ² test was applied for categorical variables, Fisher’s Exact Test for categories with cell counts < 5, and the Shapiro-Wilk test for normality of continuous variables. If *p* > 0.05, differences between two independent samples were calculated using the Student’s T-test; if *p* < 0.05, the Mann-Whitney test was used.

## Results

### Sample Characterization

Of the 22 eligible professors, 16 participated in the research, being equally distributed between the Medicine (*n* = 8) and Physiotherapy (*n* = 8) courses. Six professors were excluded for not responding to the questionnaire, according to the previously defined criteria of voluntary participation and complete responses. This resulted in a response rate of 72.7%. The participants represent the professors responsible for teaching neuroanatomy in 11 of the 18 universities in the state of Paraíba that offer Medicine and/or Physiotherapy in their undergraduate course offerings. Among these institutions, 3 exclusively offer the Medicine course, 7 only the Physiotherapy course, and another 7 have both courses in their curriculum (Table 1).

**Table 1:** Demographic and professional data of the respondents

Variable	Total (n=16)	Medicine (n = 8)	Physiotherapy (n = 8)	p-value
Age (years),	27 – 63	33-54	27-63	0.431^a^
Mean Range (SD)	42.6 (8.84)	40.8 (6.41)	44.4 (10.9)	
Self-identified gender, % (n)	Females = 37.5 (6) Males = 62.5 (10)	Females = 25 (2) Males = 75 (6)	Females = 50 (4) Males = 50 (4)	0.511^b^
Highest qualification, % (n)				
Doctoral degree	62.5 (10)	87.5 (7)	37.5 (3)	0.026*
Master’s degree	31.3 (5)	0 (0)	62.5 (5)	
Graduate (Lato-Sensu)	6.3 (1)	12.5 (1)	0 (0)	
Undergraduate	0 (0)	0 (0)	0 (0)	
Additional qualification in education, % (n)	43.8 (7)	37.5 (3)	50 (4)	1.000^b^
Additional qualification in neuroanatomy, % (n)	43.8 (7)	50 (4)	37.5 (3)	1.000^b^
Neuroanatomy teaching experience, % (n)				
Expert	62.5 (10)	50 (4)	75 (6)	0.282^b^
Proficient	31.3 (5)	50 (4)	12.5 (1)	
Trainee	6.3 (1)	0 (0)	12.5 (1)	
Beginner	0 (0)	0 (0)	0 (0)	

Values are expressed as percentages and absolute frequencies (%, n). *p < 0.05

^a^The independent samples t-test was used to compare the means between Medicine and Physiotherapy teachers (age)

^b^Fisher’s exact test was used to compare proportions between Medicine and Physiotherapy teachers (gender, academic qualifications, additional training, and teaching experience)

The 16 participants reported their highest degree in four categories: undergraduate, postgraduate (lato sensu), master’s, or doctorate. A predominance of professors with doctoral degree (62.5%) and an absence of professors with only an undergraduate degree in their respective field of activity were observed. Analyzing the courses separately, it was found that, in the sample of Medicine professors, 87.5% held a doctoral degree, while in the Physiotherapy group, master degree predominated (62.5%). Fisher’s exact test revealed a statistically significant difference between the courses regarding the academic qualification level of the professors (*p* = 0.026), indicating that the Medicine course concentrates professionals with higher formal qualifications compared to Physiotherapy (Table 1).

The professors also reported investing in complementary training in both education and neuroanatomy, showing no differences between professors from Medicine and Physiotherapy courses (*p* = 1.000) (Table 1).

Regarding the experience in teaching neuroanatomy, participants were asked to self-declare based on four levels of pedagogical competence: “beginner,” referring to professionals without prior experience; “in training,” for those acting under supervision; “proficient,” for those with consolidated teaching experience; and “specialist,” reserved for professors with deep mastery of content and extensive didactic ability. The majority classified themselves as specialists (62.5%), followed by proficient (31.3%), and a single professor identified as in training (6.3%). No participant declared themselves as a beginner. There were no differences in this aspect between the Medicine and Physiotherapy courses (*p* = 0.282) (Table 1).

## Perception of Neuroanatomy’s Relevance

All participants agreed that neuroanatomy is a fundamental curricular component for the training of healthcare students, recognizing it as an indispensable science for professional practice and that a good healthcare professional must possess solid knowledge in this area. Furthermore, there was an unanimous disagreement with the following statements: neuroanatomy is so outdated that it has no importance in contemporary medicine and other health areas; neuroanatomy is a waste of time in the healthcare professionals’ curriculum; instead of studying neuroanatomy, healthcare students should focus on clinical setting (Table 2).

**Table 2:** The relevance of neuroanatomy in the medical curriculum as perceived by the lecturing staff

Statement		Number of participants who agreed with the statement		Number of participants who disagreed with the statement	
		n	%	n	%
Neuroanatomy is an important component in the academic training of my students	**Medicine**	8	100	0	0
	**Physiotherapy**	8	100	0	0
	**Total**	**16**	**100**	**0**	**0**
Neuroanatomy is necessary for safe practice in the health sciences	**Medicine**	8	100	0	0
	**Physiotherapy**	8	100	0	0
	**Total**	**16**	**100**	**0**	**0**
Neuroanatomy has some utility in the clinical setting, but its importance may be overstated	**Medicine**	1	12.5	7	87.5
	**Physiotherapy**	2	25	6	75
	**Total**	3	18.7	13	81.3
Neuroanatomy is so old-fashioned that it has no importance in contemporary medicine and other health fields	**Medicine**	0	0	8	100
	**Physiotherapy**	0	0	8	100
	**Total**	**0**	**0**	**16**	**100**
Neuroanatomy is a waste of time in the curriculum of health professionals	**Medicine**	0	0	8	100
	**Physiotherapy**	0	0	8	100
	**Total**	**0**	**0**	**16**	**100**
Neuroanatomy needs to modernize if it is to be truly useful in the health field	**Medicine**	3	37.5	5	62.5
	**Physiotherapy**	6	75	2	25
	**Total**	9	56.3	7	43.7
A very good health professional should have a solid understanding of neuroanatomy	**Medicine**	8	100	0	0
	**Physiotherapy**	8	100	0	0
	**Total**	**16**	**100**	**0**	**0**
It is impossible to conceive quality training in the health field without a significant component of neuroanatomy	**Medicine**	6	75	2	25
	**Physiotherapy**	6	75	2	25
	**Total**	12	75	4	25
The undergraduate or postgraduate program I teach would not exist without neuroanatomy	**Medicine**	5	62.5	3	37.5
	**Physiotherapy**	5	62.5	3	37.5
	**Total**	10	62.5	6	37.5
Only a limited knowledge of neuroanatomy is necessary for safe practice in the health field	**Medicine**	1	12.5	7	87.5
	**Physiotherapy**	1	12.5	7	87.5
	**Total**	2	12.5	14	87.5
Rather than studying neuroanatomy, health students should focus on clinical settigns	**Medicine**	0	0	8	100
	**Physiotherapy**	0	0	8	100
	**Total**	**0**	**0**	**16**	**100**
Without knowledge of neuroanatomy, the health professional is of limited effectiveness	**Medicine**	7	87.5	1	12.5
	**Physiotherapy**	7	87.5	1	12.5
	**Total**	14	87.5	2	12.5

Table 2 presents the distribution of participants’ responses to agreement-based statements. Responses were collected as binary options (“agree” or “disagree”), and the table displays the number (n) and percentagem (%) of lecturers in each group selecting each option

Other distinct trends in perceptions between Medicine and Physiotherapy professors were observed. The statement that neuroanatomy “has some clinical utility, but its importance may be overestimated” had greater agreement among Physiotherapy professors (25%) than Medicine professors (12.5%). Furthermore, the idea that neuroanatomy “needs to modernize” was supported by 75% of Physiotherapy professors, compared to 37.5% of Medicine professors. On the other hand, there was total agreement among the groups (75%) regarding the impossibility of conceiving quality training without neuroanatomy, as well as the relevance of the discipline for the existence of their programs (62.5%), the insufficiency of limited knowledge (87.5%), and the negative impact of the absence of neuroanatomy on professional practice (87.5%), revealing a shared perception of its structural importance in health education (Table 2).

## Teaching Resources and Methodologies

Regarding the literature used by professors, Angelo Machado’s Functional Neuroanatomy [16] was the most utilized work both in class, as mandatory bibliography, and as a complementary recommendation, standing out among other references, being cited 9 and 5 times, respectively. Sobotta’s Atlas of Human Anatomy [17] was also cited with relevant frequency, being mentioned 5 times in total, considering its use as mandatory or complementary literature. Furthermore, other references, such as Moore [18], Netter [19], Gray [20], Snell [21], Prometheus [22], Meneses [23], and Yokochi [24], appeared with less recurrence in citations regarding the use of these literatures.

Concerning complementary pedagogical resources, the majority of professors reported making support materials available (87.5%) and recommending YouTube videos (68.7%). Additionally, e-books and scientific articles were indicated by approximately 62.5% of the participants. The recommendation of e-books also appears to be greater among Medicine professors (43.7% versus 18.7%). The use of informal sources, such as blogs and gray literature, was limited (6.2%), suggesting that lecturers tend to prioritize more formal and structured academic materials (Table 3).

**Table 3:** Materials used in class and recommendations by the lecturing staff

Teaching resources		Frequency of recommendation
Provide slides or material of support, % (n)	**Medicine**	43.7 (7)
	**Physiotherapy**	43.7 (7)
	**Total**	**87.4 (14)**
Recommends videos on Youtube, % (n)	**Medicine**	31.2 (5)
	**Physiotherapy**	37.5 (6)
	**Total**	**68.7 (11)**
Recommends websites from Anatomy, % (n)	**Medicine**	25.0 (4)
	**Physiotherapy**	18.7 (3)
	**Total**	**43.7 (7)**
Recommends the use of e-books, % (n)	**Medicine**	43.7 (7)
	**Physiotherapy**	18.7 (3)
	**Total**	**62.4 (10)**
Recommends blog articles (grey literature), % (n)	**Medicine**	6.2 (1)
	**Physiotherapy**	0.0 (0)
	**Total**	**6.2 (1)**
Recommends scientific journal articles, % (n)	**Medicine**	37.4 (6)
	**Physiotherapy**	25.0 (4)
	**Total**	**62.4 (10)**
Recommends apps on electronic devices, % (n)	**Medicine**	25.0 (4)
	**Physiotherapy**	25.0 (4)
	**Total**	**50.0 (8)**

Table 3 summarizes the frequency with which lecturers selected each teaching resource in a multiple-response checklist question. Each option functioned as an independent binary item (“selected” vs. “not selected”), and the table reports the percentage and number of lecturers who selected each resource

## Adopted Teaching Methodologies

Concerning teaching methodologies, all participants reported addressing the contents of the spinal cord, brainstem, and diencephalon. Other topics frequently included in classes were cranial nerves, meninges, telencephalon, limbic system, and reticular formation, cited by 94% of respondents. The use of anatomical specimens, whether cadaveric or models, was recorded for these topics, with variations between 42.9% and 64.3%. Dissection practices, however, were less frequent, appearing in up to 42.9% of cases, depending on the content (Table 4). When comparing the collected responses regarding the methodologies used by Medicine and Physiotherapy professors, there was no difference between these groups (*p* > 0.05) concerning the performance of practical classes, the use of cadavers or anatomical models in class, and also in the performance of dissection as a teaching methodology.

When questioned about the time allocated to each content and its adequacy, respondents indicated that the content of nervous system histology does not have adequate time to be addressed in the nervous system anatomy discipline, with only 37.5% stating that sufficient time is allocated. On the other hand, contents such as the spinal cord, brainstem, cranial nerves, among others were the topics that presented the highest rates of agreement among participants regarding the adequacy of time allocated to their approach in neuroanatomy teaching, with 81.2% (Table 4). When comparing the responses regarding this topic between Medicine and Physiotherapy professors, there were no differences between these groups (*p* > 0.05).

**Table 4:** Theme, type of class and methodology adopted by the lecturing staff

Class topic	Teaches the Content	Conducts Practical Sessions	Use of Cadaveric Parts or Models	Conducts Dissection Sessions	Time considered adequate
	%	% (n)	% (n)	% (n)	% (n)
Development of the NS	75	12.5 (2)	0	0	56.3 (9)
Histology of the NS	31.2	6.2 (1)	0	0	37.5 (6)
Spinal cord	100	81.2 (13)	64.3 (9)	28.6 (4)	81.3 (13)
Brainstem	100	87.5 (14)	64.3 (9)	28.6 (4)	81.3 (13)
Cranial Nerves	94	87.5 (14)	50 (7)	21.4 (3)	81.3 (13)
Diencephalon and Pituitary	100	87.5 (14)	51 (7)	28.6 (4)	81.3 (13)
Hemispheres, LS, RF	94	62.5 (10)	42.9 (6)	42.9 (6)	50.0 (8)
Autonomic system	75	31.2 (5)	42.9 (7)	7.1 (1)	62.5 (10)
Ventricular system	82	62.5 (10)	57.1 (8)	21.4 (3)	75.0 (12)
Meninges	94	68.8 (11)	57.1 (9)	14.3 (2)	81.3 (13)
Blood vessels	88	62.5 (10)	57.1 (10)	14.3 (2)	81.3 (13)

Table 4 presents the percentage and number of lecturers who selected each option in a set of multiple-response items about course content and teaching methods, along with categorical responses regarding the adequacy of class time (“adequate,” “inadequate,” or “not applicable”)

*NS *nervous system

*LS* limbic system

*RF *reticular formation

## Discussion

The results show that most professors (93.8%) have strong academic background and extensive experience in teaching the subject. However, a significant difference was found between the qualification levels of professors in Medicine and Physiotherapy programs. While the majority of Medicine professors hold the doctorate degree, the Physiotherapy faculty predominantly hold a masters degree, a pattern that reflects national trends, wherein the proportion of doctoral degree holders in Medicine is thirteen times higher than in Physiotherapy [25]. Thus, while our study did not assess teaching quality directly, differences in faculty qualification may influence instructors’ pedagogical perspectives and the approaches they feel prepared to adopt, particularly in complex fields such as neuroanatomy [26, 27].

All participants recognized neuroanatomy as an essential component of healthcare training, unanimously rejecting the idea that it is an outdated or irrelevant discipline. This recognition is meaningful, as teaching perspectives reflect the set of beliefs and intentions that shape and legitimize methodological choices [26]. Thus, when a teacher underestimates the relevance of neuroanatomy, this perception may be indirectly conveyed to students through pedagogical practices [14]. At the same time, exposing students to an excessive level of detail—based on the expectation that they should reach the same theoretical mastery as the instructor—can contribute to cognitive overload and has been associated in the literature with increased difficulty and negative attitudes toward neuroanatomy, sometimes described as neurophobia [14, 28]. It is important to note, however, that our study did not measure student outcomes; therefore, any links to neurophobia should be interpreted as theoretical and grounded in prior evidence, not as findings from our data.

Practical lessons using anatomical specimens, whether cadaveric or models, were reported in only about half of the classes (42.9% to 64.3%, depending on the topic). Although this proportion is not unexpected for resource-limited settings, it suggests that access to traditional practical activities remains constrained in several institutions. Dissection was even less frequent (7.1% to 42.9%), likely reflecting both the intrinsic complexity of dissecting neural structures and the limited availability of cadaveric material. These constraints are consistent with reports from other regions where logistical barriers—such as high maintenance costs, limited laboratory staff, and restricted body donation programs—have reduced opportunities for hands-on anatomy teaching. Nevertheless, the literature still positions cadaveric work as a valuable component of anatomical education, particularly for long-term retention and three-dimensional understanding [29].

Regarding teaching strategies, the strong preference for traditional textbooks may reflect confidence in long-established, peer-reviewed materials, but it may also indicate that these resources remain the most familiar and culturally ingrained tools among lecturers. Although 68.7% of participants reported using online videos as supplementary material, the adoption of more advanced technological tools was not equally widespread, suggesting that easily accessible digital resources are more readily incorporated into teaching routines than tools requiring formal training or institutional support. The cautious recommendation of online materials—particularly the low endorsement of blogs and grey literature—aligns with global findings showing that anatomy educators tend to prioritize curated and evidence-based resources, even when integrating digital components [30].

While many studies advocate for multimodal approaches that combine technology, models, and hands-on activities [31], other authors describe skepticism among faculty who question whether the pedagogical benefits of innovative technologies justify the time and effort required for their adoption. Empirical evidence suggests that tools such as 3D models and augmented reality can enhance the spatial visualization of neural structures [32–35], while dissection continues to provide a deep three-dimensional understanding and remains associated with long-term knowledge retention [30]. Such multimodal strategies enable students to develop refined spatial perception of neuroanatomical structures, their interrelationships, and individual variations—competencies essential for accurate diagnosis and safe clinical practice [36]. These contrasting perspectives also help contextualize the paradox observed in our sample: although many professors describe themselves as pedagogically “proficient,” their teaching practices remain relatively narrow, likely reflecting accumulated experience within traditional frameworks rather than sustained engagement with a broad range of instructional technologies. Together, these factors demonstrate that modernization in neuroanatomy teaching is not uniformly embraced and is shaped by local academic cultures, professional identities, and institutional priorities.

The perception of the need for innovation also differed markedly between the two groups: 75% of Physiotherapy lecturers indicated that neuroanatomy teaching should be modernized, compared to 37.5% of Medicine lecturers. Rather than repeating patterns already discussed, this contrast suggests that the programs may operate under distinct pedagogical cultures. Physiotherapy curricula in Brazil often emphasize applied and skills-oriented instruction, which may foster greater openness to integrating new teaching strategies and technologies—although this interest did not translate into higher recommendation rates of specific digital resources such as e-books, which were endorsed by only 18.7% of Physiotherapy faculty compared to 43.7% of Medicine faculty. Medicine programs, in contrast, tend to rely on more traditional academic structures and may face greater institutional rigidity, heavier curricular loads, or stronger adherence to established pedagogical identities—all factors that can make modernization less salient or more difficult to implement. As noted in a European study [37], programs that successfully balance technological tools with traditional approaches often benefit from institutional flexibility and targeted faculty development, elements that may vary substantially between courses. These differences highlight the importance of examining how curricular organization, professional identity, and institutional expectations shape educators’ willingness to adopt new methodologies.

The perceived inadequacy of class time—since no topic reached full agreement regarding sufficiency—parallels international reports of reduced anatomy hours in contemporary curricula [38]. However, our data do not allow us to determine whether similar reductions have occurred in Paraíba. Beyond time constraints, resistance to modernization may also stem from personal and logistical factors, such as the perception that change is difficult, limited reflection on pedagogical renewal, and unfamiliarity with new educational technologies [14]. These elements can hinder the adoption of more diverse teaching strategies that integrate traditional and innovative approaches. Although such strategies have been associated in the literature with improved student engagement and reduced learning barriers, our study assessed only instructors’ perceptions; therefore, any implications for student outcomes should be interpreted with caution.

During the conduct of the study, we encountered significant challenges. Initial response rate from faculty, for example, was low, which substantially prolonged the time required to achieve a minimally representative sample size. We also clarify that the study’s pilot nature contributed to this limitation, and that future research with broader participation and expanded geographic coverage will be necessary to strengthen the external validity of the results. Furthermore, the average length of time taken to respond to the questionnaire was relatively high, given the number of questions that were addressed, which may have led to responses that may not correspond to what the teachers actually thought. Despite these limitations, to the best of our knowledge, this is the first study to characterize neuroanatomy instructors in the state of Paraíba, allowing us to analyze their perceptions, pedagogical strategies, and the challenges faced in teaching practice. Such findings contribute to a clearer understanding of the local landscape of the discipline and may inform future improvements in educational practices and the training of healthcare professionals. This consensus reinforces the idea that a solid foundation in neuroanatomy is essential for safe and effective clinical practice. However, despite the positive perception, challenges remain regarding teaching methods, the integration of educational resources, and the adequacy of the workload.

## Conclusion

This study shows that neuroanatomy instructors in Paraíba recognize the discipline as essential for the training of healthcare professionals across both Medicine and Physiotherapy. Their qualifications and commitment were evident; however, the predominance of traditional teaching methods, the limited use of cadaveric-based activities, the restricted coverage of certain topics, and the perception of insufficient class time indicate structural and pedagogical constraints that may limit opportunities for innovation. Although digital resources are increasingly used, their integration remains uneven, and openness to modernization varies between courses, suggesting that institutional culture and disciplinary traditions play an important role in shaping teaching practices.

The findings highlight several areas in which institutions may act to strengthen neuroanatomy education: (1) supporting faculty development initiatives focused on technology-enhanced teaching; (2) expanding access to durable anatomical models and low-cost digital resources; (3) improving the integration of neuroanatomy with related disciplines through coordinated curricular planning; and (4) reinforcing body donation programs to increase the availability of cadaveric materials where feasible. These actions may help enhance student engagement and provide a more balanced and contemporary learning experience. It is important to note, however, that our study assessed only instructors’ perceptions; therefore, any implications for student learning outcomes, including attitudes such as neurophobia, remain theoretical.

Future research should examine larger and more diverse populations of instructors, explore how institutional resources and curricular structures shape pedagogical decisions, and investigate how students perceive the adequacy and relevance of neuroanatomy teaching. Studies that integrate both faculty and student perspectives will be particularly valuable for informing comprehensive curricular improvements.

## Data Availability

The authors confirm that the data supporting the findings of this study are available within the article. Moreover, any other datasets generated during and/or analysed during the current study are available from the corresponding author on reasonable request.
